# Stable Isotope and Elemental Characteristics for Origin Identification of Rice from China and Thailand

**DOI:** 10.3390/plants15010042

**Published:** 2025-12-23

**Authors:** Xiaofan Xing, Fengmei Sun, Weigui Zhang, Weixing Zhang, Yongzhi Zhang, Karyne M. Rogers, Chunlin Li, Yuwei Yuan

**Affiliations:** 1 State Key Laboratory for Quality and Safety of Agro-Products, Zhejiang Academy of Agricultural Sciences, Hangzhou 310021, China; 18830328226@163.com (X.X.); zhangyz@zaas.ac.cn (Y.Z.); karynerogers@gmail.com (K.M.R.); 2 College of Agriculture, Forestry and Technology, Hebei North University, Zhangjiakou 075000, China; shiliu0616@163.com; 3 Key Laboratory of Information Traceability for Agricultural Products, Ministry of Agriculture and Rural Affairs of China, Institute of Agro-Products Safety and Nutrition, Zhejiang Academy of Agricultural Sciences, Hangzhou 310021, China; 4 China National Rice Research Institute, Hangzhou 310006, China; 13655713387@163.com (W.Z.); zhangwxcnrri@163.com (W.Z.); 5 School of Geography, Environment and Earth Sciences, Victoria University of Wellington, Wellington 6012, New Zealand

**Keywords:** rice, stable isotopes, elements, chemometrics, PLS-DA, traceability, food fraud

## Abstract

China, as the primary importer of Thailand’s high-quality rice (*Oryza sativa* L.), has an urgent need for effective origin discrimination methods between premium aromatic rice from China and Thailand to prevent origin mislabeling issues. In this study, stable isotope and elemental multivariate analysis combined with partial least squares discriminant analysis (PLS-DA) were used to build an origin traceability model for Chinese and Thai rice from different production areas. Multivariate analysis of variance revealed that Thai rice exhibited significantly higher *δ*^13^C (−26.4 ± 0.4‰) and *δ*^18^O (25.9 ± 1.1‰) values, but a significantly lower *δ*^15^N value (3.5 ± 0.8‰) compared to the three major producing regions of China. These differences are directly related to geographical and climatic factors such as latitude, precipitation, and temperature. A PLS-DA model demonstrated high performance in the classification of different Chinese indica rice and Thailand rice origins. Through cross-validation, the classification accuracy for the training set reached 97.3%. For the independent testing set, the classification accuracy was recorded to be 95.0%. Furthermore, external blind sample verification was conducted, and the classification accuracy achieved was 100%. Ca, K, Na, *δ*^18^O, Zn and *δ*^2^H were found to be important variables to discriminate between Chinese indica rice and Thai rice. Finally, for country of origin labelling claims, this rice study provides the basis for a suitable regulatory method to detect mislabeled Thai origin rice and prevent fraud.

## 1. Introduction

Globally, China ranks first for production and consumption of rice (*Oryza sativa* L.), with a total rice production of 206.6 million tons in 2023 [1]. More than 60.0% of the Chinese population use rice as their daily staple food, so the production of high-quality rice and adherence to national food safety standards is paramount to protect the health of the nation. China is also the largest importer of rice products, importing more than 3.25 billion tons of rice annually from other countries to meet the domestic market consumption demand [2]. China is reported to be the primary importer of high-quality rice from Thailand [3]. Thai indica rice is very popular among Chinese consumers for its excellent quality, unique aromatic flavor and texture. Its market price is generally high in China, with prices probably around two to three times that of Chinese indica rice [4]. Driven by economic interests, unscrupulous merchants may be prone to mislabeling domestic Chinese indica rice and pass it off as premium imported Thai indica rice to obtain a higher price. Notably, the issue of fraudulent origin labeling concerning Thai indica rice has garnered significant social concern in recent years. As highlighted by the China Central Television “3·15” Evening Gala, certain companies have been accused of misrepresenting Chinese indica rice as authentic Thai fragrant (indica) rice [5]. Such practices not only egregiously violate consumers’ rights to information and choice but also disrupt the normal functioning of the rice market. Thai and Chinese indica rice have a similar shaped grain so the average consumer cannot easily make a visual distinction between origin, although Chinese japonica rice has clear shape differences compared to indica rice, so cannot be easily sold as Thai indica rice. Rice origin mislabeling could also lead to serious food quality and safety issues if contaminants are present, causing a reduction in consumer confidence of premium rice brands from both countries. Therefore, it is important to establish a rice traceability system in order to combat illegal mislabeling or counterfeiting of high-quality Thai rice to ensure fair competition and assure consumer confidence [6].

A combination of isotope and elemental data acquired from stable isotope ratio mass spectrometry (IRMS) and inductively coupled plasma mass spectrometry (ICP-MS) is now widely recognized as the basis for agricultural product traceability systems in China [7,8]. It is also more widely used internationally for origin traceability of agricultural products such as cereals, vegetables, fruits and tea [9,10,11,12]. Research indicates that the stable *δ*^2^H and *δ*^18^O isotope values of rice from Northeast China differ significantly from those grown in the Southeastern coastal provinces of Jiangsu and Zhejiang. Moreover, significant variations in *δ*^13^C, *δ*^15^N, *δ*^2^H, and *δ*^18^O values have been observed among rice produced in Southeast China, the Yangtze River Basin, and Northeast China [13]. Wadood et al. [14]. used stable isotope techniques to successfully differentiate high-quality Indian basmati rice grown different geographical regions of Pakistan. In another study, Liu et al. [15] explored stable isotope and multi-element analyses for tracing the origin of rice from different provinces in China and other Asian countries such as Malaysia. Their work pinpointed the origin of cadmium contaminated rice found in the Chinese market and consequently their results could protect food quality and safety.

Rice quality is closely related to its geographical and climatic environment. Specific growing conditions, especially during the grain-filling period, play an important role in the flavor and yield of aromatic rice. Rice responds well to a cool climate, bright sunlight and lower soil temperatures during the rice filling period, e.g., the Wuchang region of Northeast China produces high-quality rice due to long daylight hours, large diurnal temperature differences, and fertile black soil [16]. Due to varying climatic characteristics across the Asian continent, it is possible to identify different rice production regions using stable isotopes which record different environmental and climatic conditions occurring during the growth and development of a rice crop. These characteristic variations serve to identify intrinsic geographical locations [17].

The process of isotope variation or ‘fractionation’ in plants depends mainly on geoclimatic factors, environmental conditions, cultivation practices, and photosynthetic pathways [18,19]. In addition, the elemental content of rice is also closely related to the local soil composition, humidity levels, diurnal temperature fluctuations, anthropogenic inputs such as fertilizers or air or water-borne contaminants, altitude and geographical coordinates, and provides a range of unique elemental indicators to identify agricultural products [10].

In this paper, rice samples cultivated in three geographical regions of China and five growing regions in Thailand were compared using IRMS and ICP-MS. Four isotopes (*δ*^13^C, *δ*^15^N, *δ*^2^H, *δ*^18^O) and 18 elements were determined in rice collected from different production regions. The resulting data were used to build a partial least squares discriminant analysis (PLS-DA) model to enable origin classification of rice and assess the accuracy and feasibility of assigning the production location of unknown rice samples from these origins. At the same time, the origin effects between the rice isotopes and elements are discussed in relation to known climatic and environmental factors from various regions of Thailand, as well as between China and Thailand. This study contributes a strong theoretical basis for confirming the origin of Chinese and Thai rice, with a view to providing an alternative tool to improve government regulation of rice trade, protecting premium rice market value and reducing consumer fraud.

## 2. Results

### 2.1. Stable Isotopes of Thai Rice from Five Different Regions

One-way ANOVA of Thai indica rice isotope data from five regions showed there were no significant differences (*p* > 0.05) between production regions (Table 1). Mean *δ*^13^C rice values from the five Thai regions ranged from −26.8‰ to −26.1‰, indicating similar geographical and environmental growing conditions across these regions. Roi Et had the most positive mean *δ*^13^C rice value (−26.1‰) and Ubon Ratchathani had the most negative mean *δ*^13^C value (−26.8‰). Mean *δ*^13^C rice values of the other three producing regions (Amnat Charoen, Surin, and Maha Sarakham) had intermediate values around −26.4‰. Amnat Charoen rice had a higher SD of ±0.6‰ while the other sites had similar SD’s of ±0.2‰ to ±0.3‰. Mean nitrogen stable isotope (*δ*^15^N) values ranged from 3.0‰ to 4.0‰ with SD’s between ±0.5‰ to ±0.9‰ across the five regions, respectively. Generally, the mean *δ*^15^N rice value reflects the available natural soil nitrogen and applied fertilizers used for plant uptake and growth, i.e., chemical fertilizers or organic composts or manures. Thai rice cultivation is mostly based on the application of organic fertilizers such as plant composts and fresh animal manures. From these *δ*^15^N rice values, it can be roughly inferred that rice cultivated in the five Thai study regions is cultivated using organic fertilizers that have not undergone significant denitrification [20]. Mean *δ*^2^H rice values ranged from −62.8‰ to −53.3‰, where Maha Sarakham had the most positive mean *δ*^2^H value (−53.3‰) and Surin had the lowest mean *δ*^2^H value (−62.8‰). The other sites (Amnat Charoen, Roi Et, and Ubon Ratchathani) had mean *δ*^2^H rice values around −57.0‰. All sites had similar SD values between ±4.0‰ to ±6.0‰, which continued the trend of low inter-sample isotopic variability at these sites. Mean *δ*^18^O rice values ranged from 25.1‰ to 26.7‰ for the five Thai production regions with low isotopic variability of ±1.0‰, although Roi Et samples were very similar with SD’s of ±0.5‰. The *δ*^18^O isotope trend across Thai regions differed from the *δ*^2^H trend. Samples from Amnat Charoen area had the highest mean *δ*^18^O value of 26.7‰ and Ubon Ratchathani area had the lowest mean *δ*^18^O value of 25.1‰. Mean *δ*^18^O values of Surin, Roi Et, and Maha Sarakham rice were similar (26.0‰, 25.4‰, and 26.2‰, respectively). Finally, ANOVA showed no significant differences between the four stable isotopes of rice from the five main producing regions of Thailand.

### 2.2. Elemental Contents of Thai Rice from Five Different Regions

Eighteen elements were determined in rice sourced from the five producing areas in Thailand. Most elements showed no significant differences among regions (Table 1). However, there were significant differences noted for Mg between Maha Sarakham rice (139.2 ± 32.6 mg/kg) and Surin rice (234.9 ± 86.4 mg/kg), and for K between Maha Sarakham rice (604.2 ± 62.3 mg/kg) and Amnat Charoen rice (798.9 ± 114.3 mg/kg). Similarly, Mn in Maha Sarakham rice (9.0 ± 1.8 mg/kg) and Surin rice (12.9 ± 2.4 mg/kg) also showed significant differences, while Rb was lower in Roi Et and Maha Sarakham rice and higher in Amnat Charoen rice.

Overall, the elemental characteristics of rice sampled from the five regions in Thailand were very similar, with only slight variations in four elements (all of them lower in Maha Sarakham rice) across the different regions, that are mostly related to soil geochemistry. These isotopic and elemental regional similarities provide the basis for combining all the Thai regional rice data together to be used as evidence for Thailand country of origin characterization.

### 2.3. Stable Isotope Comparison of Chinese and Thai Rice

Stable isotope data of indica and japonica rice from China’s three main producing regions shows there are regional variations, particularly for *δ*^13^C and *δ*^18^O, which reflect geographical and climatic influences (Table 2). In terms of δ^13^C, the average value of rice in the Yangtze River Basin was the lowest (−28.5 ± 0.4‰), significantly lower than that in the Southeast (−27.6 ± 0.8‰) and Northeast (−26.8 ± 0.6‰) regions, reflecting the influence of climatic factors such as temperature and photosynthetic environment on rice carbon isotope composition in different regions. For *δ*^15^N, the average value of rice in the Northeast region was the highest (5.9 ± 1.2‰), significantly higher than that in the Yangtze River Basin (4.5 ± 1.6‰) and the Southeast region (4.2 ± 1.0‰), which may be related to the long-term application of organic fertilizers and stronger denitrification processes in the soil in the Northeast region, reflecting the regulatory effect of fertilization management on nitrogen isotope characteristics in different producing regions. Regarding *δ*^2^H, the value of rice in the Yangtze River Basin was the most negative (−60.6 ± 8.7‰), followed by the Northeast region (−57.4 ± 8.7‰), and the Southeast region was the highest (−54.3 ± 3.9‰). This differentiation is mainly influenced by the hydrogen isotope composition of irrigation water in each producing region, which in turn is controlled by geographic and climatic factors such as latitude, altitude, and precipitation. For *δ*^18^O, the Northeast region showed the highest value (20.6 ± 1.6‰), followed by the Southeast region (19.7 ± 1.6‰), and the Yangtze River Basin was the lowest (18.8 ± 2.4‰). The differences similarly originate from the geographical differentiation patterns of oxygen isotopes in water sources across the producing regions, making *δ*^18^O an important indicator for rice geographical traceability.

**Figure 1:**
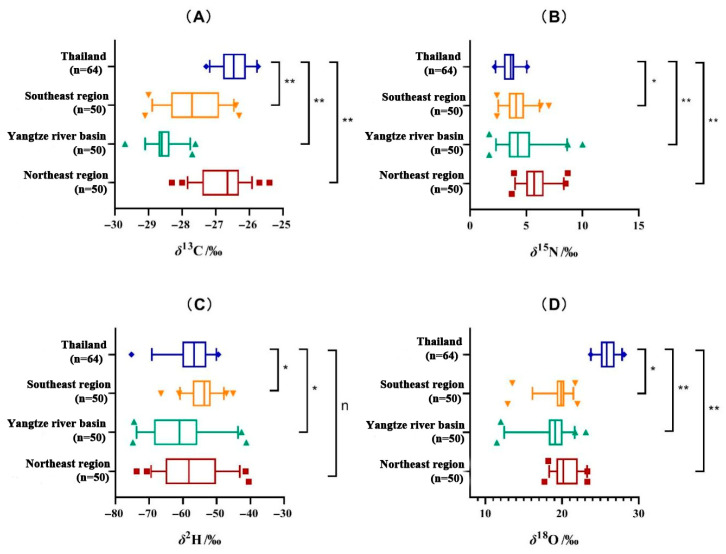
Stable isotope box plots of (**A**) *δ*^13^C, (**B**) *δ*^15^N, (**C**) *δ*^2^H and (**D**) *δ*^18^O values of rice in Thailand and three key Chinese producing regions. Note: * indicates *p* < 0.05, ** indicates *p* < 0.01, n indicates *p* > 0.05.

Rice from three key production regions in China mostly showed significant differences (*p* < 0.01) with Thai rice, especially for *δ*^13^C, *δ*^15^N and *δ*^18^O values of rice from Yangtze River Basin and Northeast China (Figure 1). The most negative mean *δ*^13^C rice value was found in Yangtze River Basin rice (−28.5 ± 0.4‰) and the Southeast producing region (−27.6 ± 0.8‰). Thai indica rice had the highest overall mean *δ*^13^C rice value of −26.4‰. Carbon isotope composition of rice is closely related to climatic conditions and hence photosynthesis. Photosynthesis rate is affected by temperature, air pressure, stomatal closure and local carbon dioxide concentrations [18]. Although the geography and growing environments of China’s Northeastern rice and Thai rice growing regions are quite different, it is hypothesized that temperate microenvironments around these regions may be the main reason for the similar carbon isotope compositions. There are also differences between mean *δ*^15^N values of Chinese and Thai rice. The mean *δ*^15^N value of Thai rice was 3.5‰ and was significantly lower than Chinese rice from all three growing regions, which had higher mean *δ*^15^N values (4.2‰ to 5.9‰). Nitrogen isotopes were highest for Northeast region rice suggesting that the organic fertilizers used to cultivate rice in China, particularly the Northeast region, had undergone more denitrification prior to application or during rice cultivation, than in Thailand. Plant *δ*^15^N values mainly affected by their nutrient sources, and the use of organic fertilizers can increase ^15^N levels in plants. Fertilizer preference patterns from different production areas can generate predictable regional differences for *δ*^15^N values. Long-term application of organic fertilizers in the Northeastern production areas may be an important factor contributing to higher *δ*^15^N values in rice [19].

Mean *δ*^2^H values of Chinese regional rice ranged from −60.6‰ to −54.3‰, with Yangtze River Basin rice having the most negative mean *δ*^2^H value (−60.6 ± 8.7‰), followed by Northeast (−57.4 ± 8.7‰) and Southeast (−54.3 ± 3.9‰) rice. The mean *δ*^2^H value of Thai rice was −57.0 ± 5.6‰ and showed differences with Yangtze Basin and Southeast rice. Mean *δ*^18^O rice values for various regions of China ranged from 18.8‰ to 20.6‰. The mean *δ*^18^O value of Northeast China rice was the most positive (20.6 ± 1.6‰), while the Yangtze River Basin region had the lowest mean rice value (18.8 ± 2.4‰), although the *δ*^18^O values of all three Chinese regions overlapped. The mean Thai *δ*^18^O rice value (25.9 ± 1.1‰) was significantly different from Southeastern China rice, and Yangtze River Basin and Northeastern rice. The *δ*^2^H and *δ*^18^O rice values are important markers of geographical difference [21]. Paddy field irrigation water isotopes are influenced by geographical distribution, such as latitude, longitude, elevation, temperature, rainfall intensity and distance inland [22], which results in a geographically distinctive hydrogen and oxygen isotope composition. In addition, rice *δ*^18^O values are also influenced by plant physiological processes such as respiration, transpiration, water use efficiency etc.

Localized variations in rice *δ*^2^H and *δ*^18^O values are mainly caused by changes in water source, i.e., different precipitation events or water from different sources such as snowmelt. Other factors include water availability and water use efficiencies during the growing season. In other research, high precipitation during the growing season is mainly associated with decreases in rice *δ*^13^C and *δ*^18^O values due to physiological water use processes; however, the *δ*^18^O values may change depending on the mean isotopic value of the precipitation event compared with the irrigation water [23]. In another rice study, a correlation between environmental variables and isotopes (*δ*^13^C, *δ*^2^H, *δ*^18^O) was found by Sheng et al. [24] Mean annual temperature, relative humidity, precipitation and sunshine hours were all significantly negatively correlated with *δ*^13^C and *δ*^18^O values. The largest negative correlation coefficient was found between *δ*^13^C and PRE (annual precipitation). SSD (sunshine hours) was significantly positively correlated with *δ*^13^C, *δ*^2^H and *δ*^18^O. RHU (relative humidity) had the strongest negative correlation with *δ*^2^H and *δ*^18^O values, with correlation coefficients of −0.40 and −0.49, respectively. Specific information regarding meteorological variables can be found in Table S1. The climate data was derived by interpolating gauged daily temperatures, precipitation, relative humidity and sunshine duration from Chinese Meteorological Administration (http://data.cma.cn/). Overall, the stable isotope characteristics of Chinese and Thai rice were mostly different, providing a good basis for using a multivariate discriminant model to differentiate between regions and countries.

### 2.4. Elemental Comparison of Chinese and Thai Rice

As shown in Table 2, there are differences in the elemental content of rice among the three main producing regions in China. Potassium (K) shows significant variation: the mean potassium content in rice from the Yangtze River Basin is the highest (1103.3 ± 371.0 mg/kg), significantly greater than that in the Northeast region (885.7 ± 127.3 mg/kg) and the Southeast region (983.3 ± 309.7 mg/kg). Copper (Cu) content is highest in the Yangtze River Basin (3.0 ± 1.7 mg/kg), followed by the Southeast region (2.6 ± 0.5 mg/kg), and lowest in the Northeast region (1.9 ± 0.4 mg/kg). Gallium (Ga), Molybdenum (Mo), Cadmium (Cd), and Barium (Ba) also show the highest concentrations in the Yangtze River Basin, while Rubidium (Rb) is lowest in the Southeast region (1.5 ± 1.2 mg/kg). These distinct elemental profiles across different producing regions provide a basis for further research on the geographical identification and traceability of rice.

**Figure 2:**
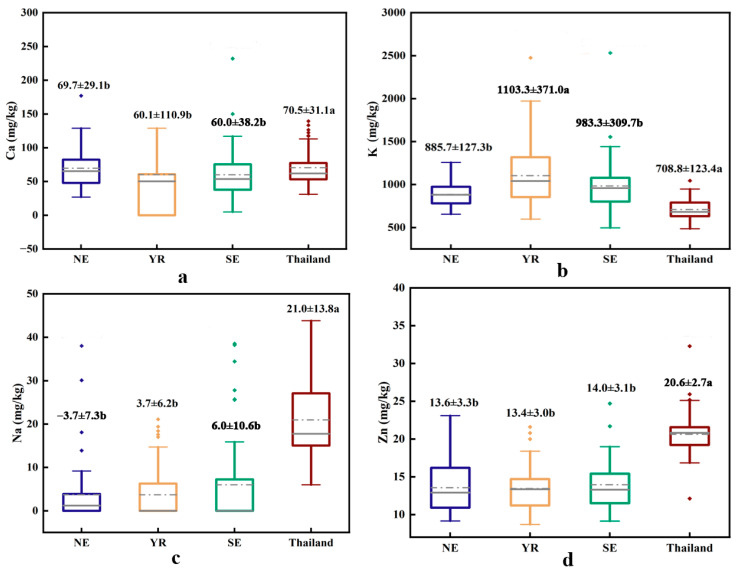
Box plots of (**a**) Ca, (**b**) K, (**c**) Na and (**d**) Zn element distributions in rice from different producing areas in China and Thailand. Note: Values are presented as mean ± standard deviation. The solid line is the median line, and the dotted line is the mean line. The horizontal line inside the box represents the median line, and the dashed line represents the mean line. Different lowercase letters indicate significant differences among groups at the 0.05 level.

Significant differences were observed in the elemental contents of rice from China and Thailand (Figure 2), with variations between Chinese production regions being less pronounced than those between the two countries. According to the Dietary Guidelines for Chinese Residents (2022) [25], key macronutrients such as sodium (Na), magnesium (Mg), K, and calcium (Ca) (essential for dietary health) exhibited distinct patterns. Notably, Thai rice had the highest mean Na content (21.0 ± 13.8 mg/kg), significantly exceeding Chinese indica rice (ranging from 3.7 ± 7.3 to 6.0 ± 10.6 mg/kg). In contrast, Mg and K levels were lower in Thai rice (182.8 ± 58.9 mg/kg and 708.8 ± 123.4 mg/kg, respectively), while Chinese indica rice from the Yangtze River Basin contained the highest concentrations (Mg: 287.2 ± 190.8 mg/kg; K: 1103.3 ± 371.0 mg/kg). No significant differences were found in Ca content between Chinese and Thai rice (60.0 ± 38.2 to 70.5 ± 31.1 mg/kg). Other trace elements iron (Fe), nickel (Ni), aluminum (Al), and lead (Pb) showed no significant differences across regions. However, Thai rice had higher chromium (Cr), zinc (Zn), Rb, and strontium (Sr) (1.8 ± 2.0 mg/kg, 20.6 ± 2.7 mg/kg, 4.7 ± 2.6 mg/kg, and 0.2 ± 0.1 mg/kg, respectively) compared to Chinese samples (Table 2). These distinct elemental profiles provide a basis for developing discriminant models using elemental signatures.

The observed differences in rice elemental contents are closely linked to soil geochemistry, which serves as the primary source of both nutrients and contaminants. Rice soils are highly modified by cultivation practices (e.g., leveling, flooding, and fertilization) and may accumulate heavy metals [26]. Notably, Thai rice soils exhibit low Pb, As, and Cd (13.70 mg/kg, 2.00 mg/kg and 0.03 mg/kg) but high Cr (91.00 mg/kg) [27], with Zn soil content (29.00 mg/kg) positively correlated with rice Zn content [28]. In contrast, Chinese indica rice soils show regional variations: Northeastern soils are rich in Mn and Fe but Zn-deficient [29], while Southeastern soils have lower Mn, Cd, and Co due to flooding-induced manganese oxide dissolution [30]. The levels of Cd and Pb detected in all rice samples in this study were compared with China’s “National Food Safety Standard for Contaminants in Foods” (GB 2762-2022) [31] and the standards established by the GB 2762 sets the maximum limit for Cd and Pb in rice at 0.2 mg/kg and 0.4 mg/kg. After moisture content conversion, the concentrations of Cd and Pb in all samples were found to be well below the regulatory limits specified in the aforementioned standards. Additionally, soil acidity affects mineral bioavailability as acidic soils enhance Cd and Zn uptake, whereas neutral/alkaline soils reduce uptake [32]. This may explain the lower Zn and Fe levels in some Chinese indica rice grown in acidic conditions [33]. Thus, soil properties and rice paddy management practices significantly contribute to elemental differences observed in Chinese and Thai rice.

### 2.5. Geographical Origin Discrimination of Rice Using PLS-DA

**Figure 3:**
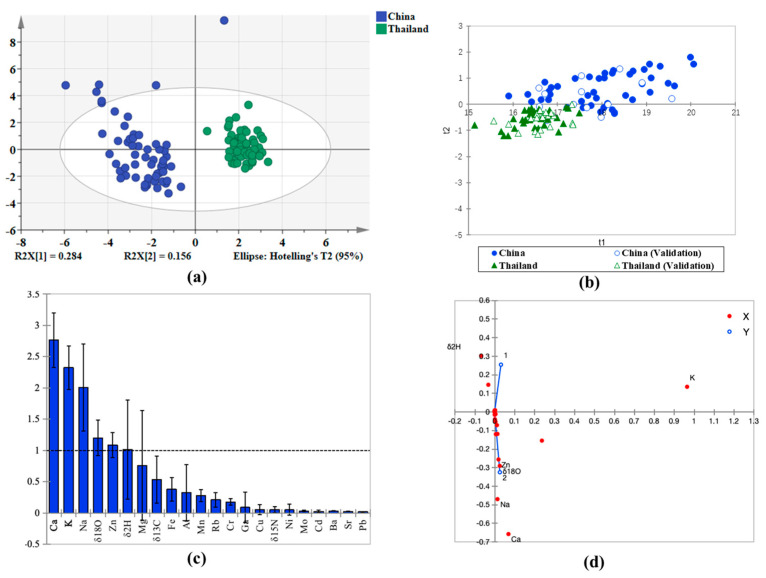
(**a**) PCA score plot of rice from China and Thailand. (**b**) PLS-DA classification scores plot of Chinese and Thai rice. (**c**) VIP diagram of Chinese and Thai rice. (**d**) Loadings plot of the PLS-DA model. Note: figure (**c**) the horizontal dashed line denotes the VIP threshold of 1: variables with bar heights above this line (VIP > 1) possess significant discriminative ability for the classification modeled in this analysis. figure (**d**) red dots (X) represent Chinese rice samples, and blue circles (Y) represent Thai rice samples. The vertical axis denotes *δ*^2^H, and the horizontal axis represents a multivariate component (labeled 1 and 2). These figures display results from a single, representative iteration of the model out of ten independent replicates.

To objectively evaluate the overall structure of the sample set and its inherent grouping trends, we initially conducted principal component analysis (PCA). As illustrated in Figure 3a, a distinct separation trend is observed between Chinese and Thai rice samples on the PCA score plot, with the first and second principal components (PC1 and PC2) accounting for 28.4% and 15.6% of the variance, respectively. This finding suggests that geographical origin significantly influences the metabolic profiles of the rice samples, while also indicating satisfactory data quality with no evident outliers. Building upon this foundation, we further employed supervised PLS-DA to enhance inter-group differences and identify key biomarkers.

In this study, a PLS-DA model was developed using the four stable isotopes and 18 elemental variables. Thai rice belongs to the indica variety and given the significant visual differences between indica and japonica rice, any rice misrepresented as Thai on the Chinese market is also of the indica type. Therefore, we developed a predictive model to distinguish between Chinese indica rice and authentic Thai rice. The PLS-DA model exhibited outstanding performance in geographical discrimination, achieving cumulative goodness-of-fit (R^2^Y) and predictability (Q^2^) values of 0.916 and 0.557, respectively. The permutation test produced highly significant results (*p* < 0.001), thereby confirming that the model’s effectiveness significantly surpasses random chance. The area under the ROC curve (AUC) is 0.995, which is significantly better than random classification (AUC = 0.5), indicating strong discriminative power. The predicted accuracy for Chinese and Thai origin rice is shown in Table 3. All values in the table are presented as the mean of 10 replicates. The training accuracy is 97.3% accuracy. Model performance was evaluated using sensitivity and specificity. In this evaluation, “Chinese rice” was defined as the positive class, and “Thai rice” was defined as the negative class. Sensitivity reflects the model’s ability to detect Chinese rice, while specificity reflects its ability to exclude Thai rice. The values for sensitivity and specificity obtained from these samples are 85.3% and 99.3%. The predictive capability of the validation model was tested with 36 rice samples (17 from China, and 19 from Thailand). All Thailand samples were correctly predicted, and sixteen out of seventeen rice samples from China were correctly identified. The model achieved a sensitivity of 81.8% for Chinese indica rice and a specificity of 100.0% for Thai rice in the prediction set. The overall discrimination accuracy of the country of origin model was 90.0% (Table S2). The PLS-DA distribution of samples is shown in Figure 3b. Thai rice samples are clustered on the bottom and Chinese indica rice samples on the top sides of the first principal component (t1), indicating that the model is able to clearly distinguish Chinese and Thai origin rice. To mitigate the impact of randomness on the accuracy of model discrimination, this study employed an independent external validation set to verify the model’s results. This validation set consisted of 10 Indica rice samples sourced from Thailand, derived from the dataset published by Wang et al. [20]. Importantly, these samples were excluded from all phases of model development, including feature selection and parameter tuning. The model achieved a perfect classification accuracy of 100% on this external set, thereby strongly demonstrating its robust discriminative power when applied to entirely unseen data. Considering the potential limitations of single-year data in assessing rice element content, we further compared data from two years (Table S3). The results indicated no significant differences in most elements between the years. Based on this, we speculate that the interannual variation in rice element content may be relatively limited.

The VIP screens the most useful variables for origin classification with VIP values > 1 leading to a higher variable classification ability. Figure 3c shows there are 6 variable which have a VIP > 1; Ca, K, Na, *δ*^18^O, Zn and *δ*^2^H, suggesting that climate related isotopes (*δ*^18^O, *δ*^2^H) and soil variables (Ca, K, Na, Zn) more strongly influence the classification characteristics for Chinese and Thai rice. Despite the low VIP values of *δ*^13^C and *δ*^15^N, existing studies have indicated significant correlations between these isotopic markers and the geographical origin of crops [34]. *δ*^15^N values are predominantly influenced by anthropogenic agricultural practices, particularly fertilization management [35]. Consequently, in the discriminant model employed, the VIP scores of these two indicators did not reach the significance threshold. Additionally, although *δ*^34^S is useful for discriminating sulfate sources, its low natural abundance in rice resulted in insufficient discriminatory power to meet the feature selection threshold. Figure 3d presents the score and loading plots of the final PLS-DA model. In the score plot, the horizontal axis distinguishes Chinese indica rice from Thai rice based on their production regions, revealing a slight overlap between the two. The findings indicate that Chinese indica rice is predominantly influenced by elements such as Mo, Ba, K, Cu, Mg, and δ^15^N. Conversely, Thai rice is primarily affected by elements including Na, Zn, *δ*^18^O, and Ca.

The PLS-DA-based model for identifying rice origins developed in this study possesses considerable practical relevance and application value. This model can accurately differentiate between Chinese and Thai rice, thereby providing reliable technical support for combating false origin labeling, enhancing market supervision, and improving product traceability. Ultimately, it contributes to ensuring the authenticity of rice products and standardizing trade practices. In the present study, an origin discrimination model for rice from different regions of China and Thailand was established using IRMS coupled with multivariate analysis techniques.

## 3. Materials and Methods

### 3.1. Sampling Experiment and Pretreatment

**Figure 4:**
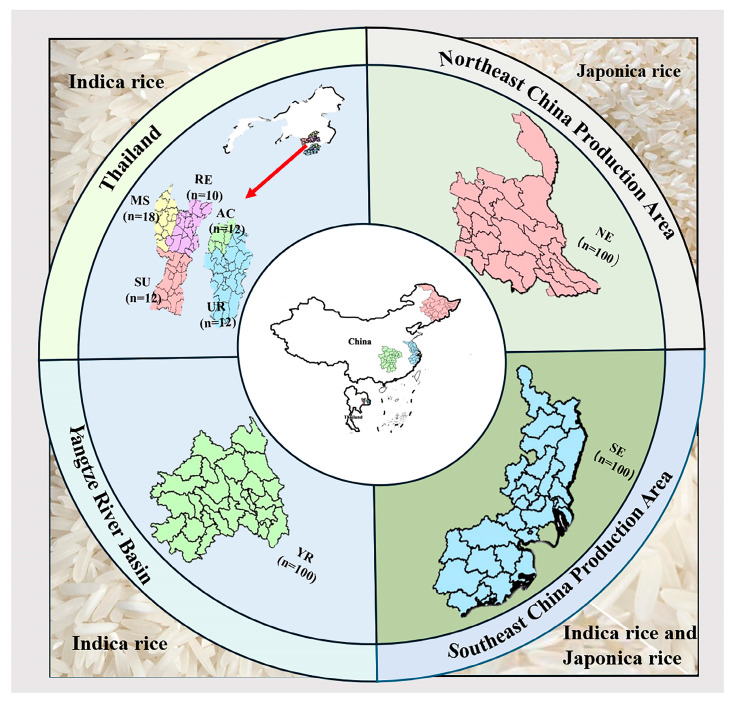
Rice sampling site locations in China and Thailand. (NE, Northeast region; YR, Yangtze River Basin; SE, Southeast region; AC, Amnat Charoen; UR, Ubon Ratchathani; SU, Surin; MS, Maha Sarakham; RE, Roi Et).

A total of 214 rice samples (500 g per sample) were collected from China (150 samples) and Thailand (64 samples). The three main rice production provinces in China are located in Northeastern China (50 samples, japonica rice) from Heilongjiang and Jilin province, the Southeastern coast (50 samples, indica rice) from Zhejiang and Jiangsu province, and the Yangtze River Basin (50 samples, japonica and indica rice) from Hunan and Hubei province. Twenty-five rice samples were collected from each province, with a total of 150 samples. A combination of grid distribution and stratified random sampling was employed to determine the sample collection sites based on the area of rice cultivation in each province. Sampling was conducted during either the harvest drying period or the storage phase, with samples collected directly from production bases or farmers’ grain storage facilities. Five major rice-producing municipalities were sampled in Thailand, including Amnat Charoen (12 samples), Ubon Ratchathani (12 samples), Surin (12 samples), Roi Et (10 samples), and Maha Sarakham (18 samples), with a total of 64 samples collected in Thailand. All samples were indica rice varieties, procured from local farmers markets in Thailand. The locations of sample collections sites from China and Thailand are shown in Figure 4.

All rice samples examined in this study were exclusively obtained from the 2019 harvest year. Rice samples were first subjected to a threshing machine to eliminate the husk, followed by processing into polished rice using a rice refiner. After drying to a constant weight in an oven at 70 °C, the samples were ground into a fine powder, passed through a 100 mesh sieve and stored at room temperature in brown vials to protect the powdered rice from light exposure.

### 3.2. Stable Isotope Analysis

For the *δ*^13^C and *δ*^15^N measurements, around 4–5 mg of each powdered rice sample was weighed in duplicate into 3 × 5 mm tin foil cups and placed in the sample tray of an elemental analyzer (Elementar vario PYRO cube, Elementar, Germany). Elemental carbon and nitrogen in the samples were converted to pure CO_2_ and N_2_ gases and then entered the isotope mass spectrometer (Isoprime 100, Isoprime, UK) for analysis. Specific instrument conditions: Elemental analyzer: helium purge flow rate of 230 mL/min; oxidation and reduction furnace temperatures were 1150 °C and 850 °C, respectively; helium flow rate of the carrier gas into the mass spectrometer was 100 mL/min. High-purity He gas was used as the carrier gas and high purity CO_2_ and N_2_ gases were used as reference gases.

The *δ*^13^C and *δ*^15^N values were calibrated relative to those of V-PDB and AIR, respectively.

For the *δ*^2^H and *δ*^18^O measurements, around 0.3 to 0.6 mg of powdered rice was weighed in triplicate into silver cups. Samples and reference materials were freeze-dried at −60 °C for three days to remove all exchangeable water and subsequently equilibrated for five days in the laboratory exposed to local atmospheric conditions prior to H and O analysis.

Samples were analyzed using a High Temperature Pyrolysis- Elemental Analyzer Isotope Ratio Mass Spectrometry (EA/HT-IRMS) The combustion furnace temperature was 1450 °C, and H_2_ and CO were used as reference gases. The *δ*^2^H and *δ*^18^O values are reported relative to those of V-SMOW.

The isotope ratio is calculated as follows:

*δ* (‰) = [(*R*_sample_/*R*_standard_) − 1] (1)

where *R*_sample_ is the ratio of the heavy to light isotope abundance in the measured sample, i.e., ^13^C/^12^C, ^15^N/^14^N, ^18^O/^16^O, ^2^H/^1^H and *R*_standard_ is the ratio of heavy to light isotope abundance in the reference standard. Isotope values are reported relative to V-PDB for *δ*^13^C, air for *δ*^15^N, and V-SMOW for *δ*^18^O and *δ*^2^H and were determined using calibrated reference materials.

Reference materials included IAEA-CH-6 (sucrose, *δ*^13^C_V-PDB_ = −10.449 ± 0.033‰), IAEA-600 (caffeine, *δ*^13^C_V-PDB_ = −27.771 ± 0.043‰, *δ*^15^N_air_ = 1.0 ± 0.2‰), IAEA-601 (benzoic acid, *δ*^18^O_V-SMOW_ = 23.14 ± 0.19‰), IAEA-602 (benzoic acid, *δ*^18^O_V-SMOW_ = 73.35 ± 0.39‰), IAEA-N-2 (ammonium sulfate, *δ*^15^N_air_ = 20.3‰ ± 0.2‰), IAEA-CH-7 (polyethylene, *δ*^2^H_V-SMOW_ = −100.3 ± 2.0‰), IAEA, B2203 (*δ*^2^H_V-SMOW_ = −25.3 ± 1.1‰), were procured from the International Atomic Energy Agency. B2155 (*δ*^15^N_Air_ = 5.94 ± 0.08‰) and B2174 (*δ*^13^C_V-PDB_ = −37.421 ± 0.017‰) were purchased from Elemental Microanalysis, UK. Throughout the continuous analysis of 180 samples, traceability of isotopic measurements to international standards was ensured by calibrating the instrument with IAEA reference materials before each batch, inserting a laboratory internal standard every 10 samples to monitor drift, applying the standard-sample bracketing method for *δ*-value calculation, and maintaining deviations of quality control samples within ±0.1‰.

### 3.3. Elemental Analysis

Powdered rice (0.1 g) was digested in triplicate in 60 mL acid-washed microwave oven vessels containing 5.0 mL HNO_3_ (65 wt%, ANPEL Laboratory Technologies Inc., Shanghai, China) and heated to 200 °C in a microwave digestion system (TOPEX, Shanghai YiYao Instruments, China) under temperature control mode for 40 min. After digestion, the vessel was cooled to room temperature, and placed on a hot plate to dry the sample until there was about 0.5 mL of liquid remaining. The residue was transferred to a 25 mL volumetric flask, where a further 0.5 mL aliquot of HNO_3_ (65 wt%) and deionized water were added to make up the remaining volume.

Eighteen elements were determined on each rice sample, including Na, Mg, K, Ca, Cr, Fe, Cu, Zn, Mn, Ni, Mo, Al, Ga, Rb, Sr, Ba, Pb, Cd, using a quadrupole tandem inductively coupled plasma mass spectrometer (Model 8900, ICP-MS/MS, Agilent, USA). Scandium, rhodium and rhenium were used as internal standard solutions (200 μg/L) to monitor and correct for instrumental drift. Elemental recovery was carefully controlled and ranged between 80% and 120%. High purity argon (99.999%) was used as the instrument carrier gas. A blank sample (a standardized solution of 2% nitric acid, consistent with the sample aliquots) was used to evaluate the background levels of Na, Mg, Al, K, Ca, Fe (0, 0.1, 0.2, 0.5, 2, and 10 ppm, respectively), Mn, Cu, Zn, Rb, Sr (0, 10, 20, 50, 200, 1000 ppb, respectively) and Cr, Ni, Mo, Ga, Ba, Pb, Cd (0, 0.1, 0.2, 0.5, 2, 10 ppb, respectively). A rice standard was digested and included for analysis after every 12 samples to evaluate precision. To verify the stability of the elemental results and data accuracy, the relative standard deviation (RSD) for each element was calculated and was less than 9.3%.

### 3.4. Model Establishment and Evaluation

Due to the significant disparities in the concentration ranges of various elements as reflected in the original spectral data (for instance, the concentration range of K is considerably lower than that of major elements such as Ca), direct modeling would lead to an over-reliance on variables with larger variances. This could hinder the model’s ability to effectively capture critical information contained within trace elements. To ensure that all variables are treated with equal importance during the modeling process and to enhance the model’s generalization capability, this study employed autoscaling preprocessing for all elemental concentration data.

The PLS-DA was carried out using XLSTAT 2019 software (Addinsoft, Lumivero USA). Relationships between isotopes, elements and origin was established using PLS-DA models, with 22 variables including four stable isotopes (*δ*^13^C, *δ*^15^N, *δ*^2^H and *δ*^18^O) and 18 elemental contents (Na, Mg, K, Ca, Cr, Fe, Cu, Zn, Mn, Ni, Mo, Al, Ga, Rb, Sr, Ba, Pb, Cd). Our study exclusively utilized visually distinguishable indica rice varieties to ensure the robustness and comparability of the model. We study collected a total of 214 rice samples, comprising 150 from China (including both japonica and indica varieties) and 64 indica samples from Thailand. Given the distinct morphological differences between japonica and indica rice, we exclusively used indica varieties for modeling to exclude varietal interference. Consequently, the model incorporated 123 indica samples (59 China and 64 Thailand). All samples were randomly divided into a training set and a validation set at a 2:1 ratio, containing 87 samples (42 Chinese and 45 Thai) and 36 samples, respectively.

To improve the robustness of the model and to ensure the statistical significance of the evaluation results, the entire modeling and evaluation process was independently conducted 10 times. To enhance the stability of the model, five-fold cross-validation was implemented during training for parameter optimization and to mitigate the risk of overfitting. Model performance was assessed using regression metrics such as R^2^ and Q^2^, alongside classification metrics including sensitivity and specificity. Furthermore, an external blind sample set (n = 10) was utilized for additional validation The Variable Importance for Projection (VIP) method was used to identify important variables useful for origin discrimination. Independent variables with VIP > 1 are considered to contribute significantly to the model

The VIP calculation formula is as follows:

(2) $${VIP}_{j}=\sqrt{\frac{k}{\sum_{h=1}^{m}r^{2}(y,c_{h})}\sum_{h=1}^{m}r^{2}(y,c_{h})w_{hj}^{2}}$$

where VIP*_j_* is the VIP value of the *j*th independent variable, k is the number of independent variables, y is the dependent variable and c_h_ is the principal component extracted from the independent variables, r (y, c_h_) is the correlation coefficient between the dependent variable and the principal component and represents the ability of the principal components to explain the dependent variable, w_hj_ is the loading of the *j*th independent variable on the *h*th principal component.

### 3.5. Data Analysis

The experimental data were analyzed using one-way ANOVA in SPSS 24 (IBM Corp., Armonk, NY, USA), with results expressed as mean ± standard deviation (Mean ± SD). To eliminate dimensional differences, all data were standardized using The Unscrambler X 10.1 software prior to modeling. Data dimensionality reduction and model construction were further implemented with tools such as MATLAB R2016a, SIMCA 14.1, and XLSTAT 2019. Finally, relevant graphs were generated using ArcMap 10.8.1 and Origin 2021 software.

## 4. Conclusions

In this study, stable isotopes (*δ*^13^C, *δ*^15^N, *δ*^2^H, and *δ*^18^O) and 18 elements (Na, Ca, Fe, Zn, Rb, Ag, and Cd, etc.) combined with PLS-DA models were used to make a clear distinction between the high-quality rice-producing regions of China and Thailand. Multi-factor ANOVA showed that regional climate and nutrient patterns had significant effects on isotope values. Regional nutrient differences contributed more significantly to change the *δ*^13^C and *δ*^15^N values, while different climatic factors contributed mostly to differences in *δ*^2^H and *δ*^18^O values. Finally, PLS-DA origin traceability models were constructed for Chinese indica rice and Thai rice. Country and regional model achieved high origin classification accuracies in both the training and testing sets (97.3% and 95.0%, respectively). This study shows the isotope and elemental modelling can provide a useful tool for regulatory authorities to discriminate Chinese indica rice and Thai rice, and ensure the labelling authenticity of imported and regional rice. Future studies will focus on increasing this database over multiple years and focus on key isotope and elemental variables (particularly heavy metals) to sustain the accuracy and capability of the model, while protecting food safety and consumer health.

## Figures and Tables

**Table 1:** Mean and standard deviations of stable isotopes and elemental contents of Thailand rice from 5 regions. Different letters denote significant differences at *p* < 0.05.

		Amnat Charoen (n = 12)	Ubon Ratchathani (n = 12)	Surin (n = 12)	Roi Et (n = 10)	Maha Sarakham (n = 18)
Stable Isotopes (‰)	*δ*^13^C	−26.4 ± 0.6 ^ab^	−26.8 ± 0.2 ^b^	−26.6 ± 0.4 ^ab^	−26.1 ± 0.2 ^a^	−26.3 ± 0.3 ^ab^
	*δ*^15^N	3.0 ± 0.5 ^a^	3.2 ± 0.9 ^a^	4.01 ± 0.7 ^a^	3.8 ± 0.7 ^a^	3.7 ± 0.9 ^a^
	*δ*^2^H	−55.9 ± 4.3 ^ab^	−56.4 ± 5.5 ^ab^	−62.79 ± 6.6 ^b^	−58.4 ± 4.4 ^ab^	−53.3 ± 3.4 ^a^
	*δ*^18^O	26.7 ± 0.9 ^a^	25.1 ± 0.8 ^a^	25.98 ± 1.0 ^a^	25.4 ± 0.5 ^a^	26.2 ± 1.3 ^a^
Elements (mg/kg)	Na (mg/kg)	23.0 ± 9.3 ^a^	16.6 ± 2.4 ^a^	15.4 ± 2.3 ^a^	15.3 ± 3.5 ^a^	29.4 ± 23.0 ^a^
	Mg (mg/kg)	205.8 ± 51.6 ^ab^	166.4 ± 40.9 ^ab^	234.9 ± 86.4 ^a^	190.8 ± 20.5 ^ab^	139.2 ± 32.6 ^b^
	Al (mg/kg)	8.4 ± 1.7 ^a^	6.9 ± 0.9 ^a^	6.7 ± 1.5 ^a^	6.7 ± 1.6 ^a^	7.5 ± 1.3 ^a^
	K (mg/kg)	798.9 ± 114.3 ^a^	700.6 ± 92.0 ^ab^	765.1 ± 169.6 ^ab^	731.0 ± 71.0 ^ab^	604.2 ± 62.3 ^b^
	Ca (mg/kg)	64.9 ± 8.8 ^a^	85.2 ± 37.9 ^a^	93.4 ± 52.8 ^a^	61.2 ± 9.9 ^a^	54.2 ± 8.4 ^a^
	Cr (mg/kg)	1.9 ± 1.8 ^a^	2.9 ± 2.4 ^a^	1.3 ± 1.4 ^a^	1.7 ± 2.7 ^a^	1.3 ± 1.7 ^a^
	Mn (mg/kg)	11.8 ± 2.2 ^ab^	10.0 ± 1.8 ^ab^	12.9 ± 2.4 ^a^	10.0 ± 2.7 ^ab^	9.0 ± 1.8 ^b^
	Fe (mg/kg)	11.1 ± 4.6 ^a^	11.3 ± 4.4 ^a^	35.0 ± 65.3 ^a^	9.4 ± 8.3 ^a^	5.8 ± 2.6 ^a^
	Ni (mg/kg)	0.3 ± 0.2 ^a^	0.2 ± 0.1 ^a^	0.3 ± 0.1 ^a^	0.2 ± 0.0 ^a^	1.4 ± 3.5 ^a^
	Cu (mg/kg)	1.7 ± 0.5 ^a^	1.7 ± 0.8 ^a^	2.1 ± 0.6 ^a^	2.1 ± 0.4 ^a^	1.5 ± 0.7 ^a^
	Zn (mg/kg)	20.7 ± 1.1 ^a^	22.2 ± 5.2 ^a^	21.0 ± 1.0 ^a^	20.9 ± 1.8 ^a^	19.2 ± 1.8 ^a^
	Ga (mg/g)	3.0 ± 2.0 ^a^	3.0 ± 1.0 ^a^	4.0 ± 1.0 ^a^	2.0 ± 1.0 ^a^	3.0 ± 1.0 ^a^
	Rb (mg/kg)	7.5 ± 3.1 ^a^	4.1 ± 1.4 ^ab^	5.5 ± 3.3 ^ab^	3.4 ± 1.1 ^b^	3.5 ± 1.2 ^b^
	Sr (mg/kg)	0.1 ± 0.0 ^a^	0.2 ± 0.1 ^a^	0.2 ± 0.0 ^a^	0.2 ± 0.1 ^a^	0.1 ± 0.0 ^a^
	Mo (mg/g)	50.0 ± 10.0 ^a^	50.0 ± 20.0 ^a^	40.0 ± 10.0 ^a^	50.0 ± 10.0 ^a^	40.0 ± 20.0 ^a^
	Cd (mg/g)	2.0 ± 1.0 ^a^	1.0 ± 1.0 ^a^	1.0 ± 0.0 ^a^	1.0 ± 1.0 ^a^	2.0 ± 3.0 ^a^
	Ba (mg/g)	80.0 ± 50.0 ^a^	60.0 ± 10.0 ^ab^	50.0 ± 10.0 ^ab^	30.0 ± 20.0 ^b^	30.0 ± 10.0 ^b^
	Pb (mg/g)	40.0 ± 10.0 ^a^	40.0 ± 10.0 ^a^	60.0 ± 30.0 ^a^	40.0 ± 10.0 ^a^	40.0 ± 10.0 ^a^

**Table 2:** Differences in isotopic and elemental characteristics of Chinese and Thai rice. Different letters denote significant differences at *p* < 0.05.

Parameters		China			Thailand (n = 64)
		Northeast (n = 50)	Yangtze River Basin (n = 50)	Southeast (n = 50)	
Stable Isotopes (‰)	*δ*^13^C	−26.8 ± 0.6 ^b^	−28.5 ± 0.4 ^d^	−27.6 ± 0.8 ^c^	−26.4 ± 0.4 ^a^
	*δ*^15^N	5.9 ± 1.2 ^a^	4.5 ± 1.6 ^b^	4.2 ± 1.0 ^bc^	3.5 ± 0.8 ^c^
	*δ*^2^H	−57.4 ± 8.7 ^ab^	−60.6 ± 8.7 ^b^	−54.3 ± 3.9 ^a^	−57.0 ± 5.6 ^ab^
	*δ*^18^O	20.6 ± 1.6 ^b^	18. 8 ± 2.4 ^c^	19.7 ± 1. 6 ^ab^	25.9 ± 1.1 ^a^
Elements	Na (mg/kg)	3.7 ± 7.3 ^b^	3.7 ± 6.2 ^b^	6.0 ± 10.6 ^b^	21.0 ± 13.8 ^a^
	Mg (mg/kg)	224.9 ± 99.9 ^ab^	287.2 ± 190.8 ^a^	229.6 ± 119.0 ^ab^	182.8 ± 58.9 ^b^
	Al (mg/kg)	4.1 ± 8.1 ^a^	4.5 ± 17.1 ^a^	3.0 ± 6.1 ^a^	7.3 ± 1.5 ^a^
	K (mg/kg)	885.7 ± 127.3 ^b^	1103.3 ± 371.0 ^a^	983.3 ± 309.7 ^b^	708.8 ± 123.4 ^c^
	Ca (mg/kg)	69.7 ± 29.1 ^a^	60.1 ± 110.9 ^a^	60.0 ± 38.2 ^a^	70.5 ± 31.1 ^a^
	Cr (mg/kg)	0.4 ± 0.3 ^b^	0.8 ± 1.1 ^b^	0.4 ± 0.3 ^b^	1. 8 ± 2.0 ^a^
	Mn (mg/kg)	13.6 ± 3.3 ^a^	10.5 ± 4.0 ^b^	11.3 ± 3.6 ^b^	10.6 ± 2.5 ^b^
	Fe (mg/kg)	8.6 ± 10.5 ^a^	13.6 ± 19.2 ^a^	9.9 ± 10.5 ^a^	13.9 ± 28.6 ^a^
	Ni (mg/kg)	0.7 ± 0.7 ^a^	0.8 ± 0.6 ^a^	0.8 ± 0.5 ^a^	0.6 ± 1.9 ^a^
	Cu (mg/kg)	1.9 ± 0.4 ^b^	3.0 ± 1.7 ^a^	2.6 ± 0.5 ^a^	1.8 ± 0.6 ^b^
	Zn (mg/kg)	13.6 ± 3.3 ^b^	13.4 ± 3.0 ^b^	14.0 ± 3.1 ^b^	20.6 ± 2.7 ^a^
	Ga (mg/g)	28.0 ± 28.0 ^b^	97.0 ± 62.0 ^a^	35.0 ± 35.0 ^b^	3.0 ± 1.0 ^c^
	Rb (mg/kg)	1.9 ± 1.0 ^bc^	2.9 ± 2.4 ^b^	1. 5 ± 1.2 ^c^	4.7 ± 2. 6 ^a^
	Sr (mg/kg)	0.1 ± 0.2 ^b^	0.1 ± 0.1 ^b^	0.1 ± 0.1 ^b^	0.2 ± 0.1 ^a^
	Mo (mg/g)	320.0 ± 110.0 ^b^	510.0 ± 190.0 ^a^	480.0 ± 220.0 ^a^	50.0 ± 10.0 ^c^
	Cd (mg/g)	11.0 ± 26.0 ^b^	243.0 ± 356.0 ^a^	46.0 ± 45.0 ^b^	1.0 ± 2.0 ^b^
	Ba (mg/g)	120.0 ± 110.0 ^b^	300.0 ± 220.0 ^a^	100.0 ± 120.0 ^b^	50.0 ± 30.0 ^b^
	Pb (mg/g)	30.0 ± 40.0 ^a^	10.0 ± 20.0 ^a^	80.1 ± 280.0 ^a^	40.0 ± 20.0 ^a^

**Table 3:** PLS-DA classification model performance for 10 repeats.

Sample Set	Origin	Sample Number	Accuracy
Training set (70%)	Chinese indica rice (Sensitivity)	42	95.3%
	Thai rice (Specificity)	45	99.3%
	All	87	97.3%
Testing set (30%)	Chinese indica rice (Sensitivity)	13	90.0%
	Thai rice (Specificity)	23	100.0%
	All	36	95.0%
External Validation		10	100%

## Data Availability

All data are presented in this report.

## Supplementary Materials

The following supporting information can be downloaded at: https://www.mdpi.com/article/10.3390/plants15010042/s1, Table S1: Mean annual meteorological data for regional rice growing regions in China and Thailand. Data sourced from China Meteorological Administration in 2017 (Daily Meteorological Data. 2017, Dataset, http://data.cma.cn/); Table S2: PLS-DA modeling of indica rice origin China and Thailand using 123 variables. The accuracy rates of the test set and prediction set from ten modeling iterations, respectively, and the corresponding validation metrics, including Q^2^, R^2^, sensitivity, and specificity; Table S3: 2019 and 2021 Raw Rice Element Data; Figure S1: ROC Curve of the PLS-DA Model.

## Abbreviations

The following abbreviations are used in this manuscript:

PLS-DA	partial least squares discriminant analysis
IRMS	isotope ratio mass spectrometry
ICP-MS	inductively coupled plasma mass spectrometry
RSD	relative standard deviation
VIP	Variable Importance for Projection

## References

[1] Xu C.C., Ji L., Chen Z.D., Fang F.P. (2022). Analysis of China’s Rice Industry in 2022 and the Outlook for 2023. Rice Sci..

[2] General Administration of Customs of the People’s Republic of China (2020). China Customs Statistical Yearbook.

[3] Xu C.C., Ji L., Chen Z.D., Fang F.P. (2023). Analysis of China’s Rice Industry in 2023 and the Outlook for 2024. Rice Sci..

[4] Chan-in P., Jamjod S., Prom-u-thai C., Rerkasem B., Russell J., Pusadee T. (2024). Application of Silicon Influencing Grain Yield and Some Grain Quality Features in Thai Fragrant Rice. Plants.

[5] CCTV 3·15 Gala Exposure: The Truth Behind ‘Thai’ Fragrant Rice. China Central Television. https://tv.cctv.cn/2023/03/15/VIDAmWrH1iEEkbQVz1OW3fJX230315.shtml.

[6] Kukusamude C., Puripunyavanich V., Kongsri S. (2023). Combination of light stable isotopic and elemental signatures in Thai Hom Mali rice with chemometric analysis. Food Chem. X.

[7] Suzuki Y. (2022). Achieving Food Authenticity and Traceability Using an Analytical Method Focusing on Stable Isotope Analysis. Anal. Sci. Adv..

[8] Yang X.T., Li Y.L., Zhao S.L., Zhang P., Zhao Y. (2024). Geographical origin authentication of agricultural products in the China–EU Geographical Indications Agreement: A comprehensive review of Chinese products. Trends Food Sci. Technol..

[9] Sinkovič L., Ogrinc N., Potočnik D., Meglič V. (2022). Isotope Fingerprints of Common and Tartary Buckwheat Grains and Milling Fractions: A Preliminary Study. Foods.

[10] Liu X., Liu Z., Qian Q., Song W., Rogers K.M., Rao Q., Wang S., Zhang Q., Shao S., Tian M. (2020). Isotope chemometrics determines farming methods and geographical origin of vegetables from Yangtze River Delta Region, China. Food Chem..

[11] Brombin V., Mistri E., Bianchini G. (2022). Multi stable isotope ratio analysis for the traceability of northern Italian apples. Food Chem. X.

[12] Huang Q., Xia H.T., Zhang T.C., Li S., Wang Y.H., Yu X.F., Zhao H.F. (2024). Traceability of Guizhou green tea production areas based on multi-element characteristics combined with biochemical components. Int. J. Food Sci. Technol..

[13] Chung I.M., Kim J.K., Prabakaran M., Yang J.H., Kim S.H. (2016). Authenticity of rice (*Oryza sativa* L.) geographical origin based on analysis of C, N, O and S stable isotope ratios: A preliminary case report in Korea, China and Philippine. J. Sci. Food Agric..

[14] Wadood S.A., Li C.L., Nie J., Rogers K.M., Mei H.Y., Zhang Y.Z., Shah I.U., Qamar A., Yuan Y.W. (2024). Stable isotopic fingerprinting of authentic basmati rice from Pakistan. Food Control.

[15] Liu Z., Zhang W.X., Zhang Y.Z., Chen T.J., Shao S.Z., Zhou L., Yuan Y.W., Xie T.Z., Rogers K.M. (2019). Assuring food safety and traceability of polished rice from different production regions in China and Southeast Asia using chemometric models. Food Control.

[16] Lin L., Wu J., Liu C., Yu C., Liu Z., Yuan Y. (2020). Study on hyperspectral identification method of rice origin in Northeast/non-Northeast China based on conjunctive model. Spectrosc. Spectr. Anal..

[17] Wang J.S., Chen T.J., Zhang W.X., Zhao Y., Yang S.M., Chen A.L. (2020). Tracing the geographical origin of rice by stable isotopic analyses combined with chemometrics. Food Chem..

[18] Liu W.W., Chen Y., Liao R.X., Zhao J., Yang H., Wang F.H. (2021). Authentication of the geographical origin of Guizhou green tea using stable isotope and mineral element signatures combined with chemometric analysis. Food Control.

[19] Jiao F., Zhang D., Chen Y., Wu J., Zhang J. (2023). Effects of Long-Term Straw Returning and Nitrogen Fertilizer Reduction on Soil Microbial Diversity in Black Soil in Northeast China. Agronomy.

[20] Wang H.C., Liu Z.D., Ma L., Li D.D., Liu K.L., Huang Q.H., Zhao B.Z., Zhang J.B. (2021). Denitrification Potential of Paddy and Upland Soils Derived from the Same Parent Material Respond Differently to Long-Term Fertilization. Front. Environ. Sci..

[21] Cui L.L., Chen H., Chen Z.P., Yuan Y.W., Han S.L., Fu Y.N., Hou H.W., Hu Q.Y. (2023). Geographical origin classification of tobacco by stable isotope and multi-elemental analysis in combination with chemometric methods. Microchem. J..

[22] Zhang M.L., Li C.C., Liu Y.M., Zhang Y.Z., Nie J., Shao S.Z., Mei H.Y., Rogers K.M., Zhang W.X., Yuan Y.W. (2024). Effects of Water Isotope Composition on Stable Isotope Distribution and Fractionation of Rice and Plant Tissues. J. Agric. Food Chem..

[23] Choi S.H., Shin W.J., Bong Y.S., Lee K.S. (2022). Effects of climate factors on spatiotemporal variation in carbon and oxygen isotope ratios in Korean rice. J. Food Compos. Anal..

[24] Sheng M.L., Zhang W.L., Nie J., Li C.L., Zhu A.X., Hu H., Lou W.D., Deng X.F., Lyu X.N., Ren Z.Q. (2022). Predicting isoscapes based on an environmental similarity model for the geographical origin of Chinese rice. Food Chem..

[25] Chinese Nutrition Society (2022). Dietary Guidelines for Chinese Residents.

[26] Lin C.Q., Huang H.B., Hu G.R., Yu R.L., Hao C.L., Lin Y. (2019). Assessment of the Speciation and Pollution of Heavy Metals in Paddy Soils from the Jiulong River Basin. Environ. Sci..

[27] Prakongkep N., Suddhiprakarn A., Kheoruenromne I., Smirk M., Gilkes R.J. (2008). The geochemistry of Thai paddy soils. Geoderma.

[28] Zheng S., Xu C., Lv G.H., Shuai H., Zhang Q., Zhu Q.H., Zhu H.H., Huang D.Y. (2023). Foliar zinc reduced Cd accumulation in grains by inhibiting Cd mobility in the xylem and increasing Cd retention ability in roots. Environ. Pollut..

[29] Zhang Z.H., Dai H.M., Song Y.H. (2022). Geochemical characteristics of some soil trace elements in the Wuyuer River Basin, Heilongjiang Province. Geophys. Geochem. Explor..

[30] Wong S.C., Li X.D., Zhang G., Qi S.H., Min Y.S. (2002). Heavy metals in agricultural soils of the Pearl River Delta, South China. Environ. Pollut..

[31] (2022). National Food Safety Standard for Contaminants in Foods.

[32] Kabata-Pendias A. (2004). Soil–plant transfer of trace elements—An environmental issue. Geoderma.

[33] Min F.F., Wang X.Y., Li L., Xin Z.J., Li X.H., Zhang T., Sun X.Y., You H.L. (2024). Effects of silicate stabilizers on cadmium reduction and the quality of rice grains in acidic paddy soil. Sci. Rep..

[34] Bilgin A.K., Cenbiz M.F., Karakaş-Budak B., Gümüs C., Alırız Kılıç S., Perinçek F., Basançelebi O., Sezik E., Certel M. (2023). Elemental compositions and stable isotope signatures for determining the geographical origin of salep orchids collected from different regions. J. Appl. Res. Med. Aromat. Plants.

[35] Jing K., Shi W., Liu L., Wang Y. (2023). Assessment of nitrogen fertilization in cotton/soybean intercropping using the ^15^N isotope dilution method. Soil Use Manag..

