# Supplementary figures and images for: Stable Isotope and Elemental Characteristics for Origin Identification of Rice from China and Thailand
Source: Plants (Basel). 2025 Dec 23;15(1):42. doi: 10.3390/plants15010042 (PMC12787399; doi:10.3390/plants15010042)

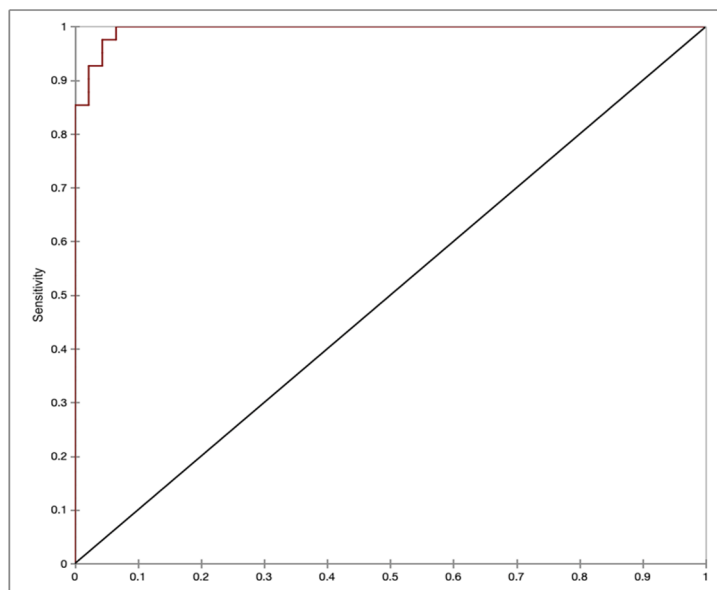

Figure S1 ROC Curve of the PLS-DA Model

Supplement: Supplementary file 1 [file plants-15-00042-s001.zip › Figure S1 .pdf]
